# Type 2 diabetes is associated with the accumulation of senescent T cells

**DOI:** 10.1111/cei.13344

**Published:** 2019-07-08

**Authors:** E. Y. M. Lau, E. C. Carroll, L. A. Callender, G. A. Hood, V. Berryman, M. Pattrick, S. Finer, G. A. Hitman, G. L. Ackland, S. M. Henson

**Affiliations:** ^1^ Barts and The London School of Medicine and Dentistry William Harvey Research Institute, Queen Mary University of London London UK; ^2^ Barts and The London School of Medicine and Dentistry Blizard Institute, Queen Mary University of London London UK; ^3^ Barts Health NHS Trust London UK

**Keywords:** ageing, inflammation, senescent, T cell, type 2 diabetes

## Abstract

Type 2 diabetes is a global health priority, given that it is driven, in part, by an ageing population, the role of immune senescence has been overlooked. This is surprising, as the functional impairments of senescent T cells show strong similarities to patients with hyperglycaemia. Immune senescence is typified by alterations in T cell memory, such as the accumulation of highly differentiated end‐stage memory T cells, as well as a constitutive low‐grade inflammation, which drives further immune differentiation. We show here in a preliminary study that people living with type 2 diabetes have a higher circulating volume of senescent T cells accompanied with a higher level of systemic inflammation. This inflammatory environment drives the expression of a unique array of chemokine receptors on senescent T cells, most notably C‐X‐C motif chemokine receptor type 2. However, this increased expression of migratory markers does not translate to improved extravasation owing to a lack of glucose uptake by the T cells. Our results therefore demonstrate that the presence of senescent T cells has a detrimental impact on immune function during type 2 diabetes.

## Introduction

Type 2 diabetes (T2D) is a metabolic disorder characterized by an impaired glucose homeostasis due to insulin resistance, associated with a chronic low‐grade systemic inflammation 1. The global disease burden in 2014 was estimated to be approximately 8% of the adult population and has been predicted to rise steeply in the coming years 2. Common co‐morbidities of T2D include increased risk of cardiac disease, alongside poor wound healing and premature onset of various age‐related disorders 1, 3, 4. Furthermore, the immense burden of mortality which accompanies T2D and its complications was estimated to account for 6.8% of global deaths in 2010 5. The pandemic nature, alongside its discernible morbidity, exposes the mammoth threat to public health that T2D truly poses.

Early studies proposed that dysfunctional innate immunity was the cause of the chronic low‐grade inflammation seen in T2D, leading many groups to concentrate on the contribution of the macrophage to diabetic disease advancement 6. However, more recently there has been heightening support advocating the role of T cell dysfunction in the pathological course of T2D. The exact mechanism in which this systemic inflammation is potentiated is still unclear; however, one probable candidate is thought to be cellular senescence 9. Cellular senescence forms a pillar of biological ageing and is implicated in various age‐related disorders, including T2D 7. Cellular senescence has been well described in fibroblast models and demonstrates cellular characteristics such as a stable cell cycle arrest, up‐regulation of cyclin‐dependent kinase inhibitors, marked alterations in cellular pathway activity and transcriptional changes, including the production of a proinflammatory senescence‐associated secretory phenotype (SASP) 8.

Senescence‐associated changes are typified in the T cell compartment by the accumulation of highly differentiated end‐stage memory T cells, as well as a constitutive low‐grade inflammation 9. Furthermore, recent evidence implicates the systemic milieu seen in T2D as a senescence‐promoting factor as well as potentiating diabetic disease progression and inflammation 7. This theory has also gained traction in studies observing the effects of metformin, where both murine 10 and human studies 11 have shown a survival advantage with metformin treatment, theorized to be a consequence of inhibiting the SASP production by modulating the activation of the nuclear factor kappa B (NF‐κB) proinflammatory pathway 12. Senescent T cells can be defined on the basis of surface marker expression, such as the loss of the co‐stimulatory molecules CD27 and CD28 or the homing marker CCR7 and the re‐expression of CD45RA, together with elevated levels of viral‐specific makers CD57 and killer cell lectin‐like receptor G1 (KLRG1) 13, 14. While senescent T cells show proliferative impairment their ability to secrete proinflammatory cytokines such as interferon (IFN)‐γ and tumour necrosis factor (TNF)‐α are retained 14, 15, 16.

The role played by senescent T cells in T2D has been overlooked. This is surprising, as the immune impairments inherent to ageing also resemble those of chronic hyperglycaemia: poor control of infections and reduced vaccination responses together with elevated inflammatory activity 17, 18. We show here in a preliminary study that people living with T2D have an increased number of senescent T cells in both the CD4^+^ and CD8^+^ T cell compartments. These senescent T cells show impaired migratory capacity despite having an up‐regulated chemokine receptor expression. Therefore, our results demonstrate that, rather than being a benign presence, the presence of senescent T cells have a detrimental impact on immune function during T2D.

## Materials and methods

### Blood sample collection, isolation and cell culture

Heparinized peripheral blood samples were taken from elderly healthy volunteers (age range = 55–73 years, mean age = 63 ± 1·5 years) and people living with T2D recruited from the DARE (Diabetes Alliance for Research in England) database (https://www.qmul.ac.uk/blizard/research/research-groups/barts-diabetes--obesity-research-group/dare/) (age range = 55–77 years, mean age = 63 ± 1 year). Peripheral blood mononuclear cells (PBMCs) were isolated using Ficoll Hypaque (Amersham Biosciences, Little Chalfont, UK). All samples were obtained in accordance with the North East‐York Research Ethics Committee 16/NE/0073.

Leucocyte data were obtained from the VISION‐UK study (MREC:10/WNo03/25) 19. Leucocyte subsets were measured using a Sysmex XE2100 analyser (Sysmex, Milton Keynes, UK).

### Flow cytometric analysis and cell sorting

Flow cytometric analysis was performed using the following antibodies: CD4 PECF594 (RM4‐5) from BD Biosciences (Oxford, UK) and CD8 peridinin chlorophyll (PerCP) (SK1), CD45RA BV605 (HI100), CD27 BV421 (O323), CCR7 phycoerythrin cyanin 7 (PECy7) (G043H7), CXCR2 fluorescein isothiocyanate (FITC) (5E8) and CX3CR1 (K0124E1) from BioLegend (London, UK), together with a live/dead fixable near‐IR stain (Invitrogen, Basingstoke, UK). A total of 1 × 10^6^ PBMCs were incubated with antibody for 15 min at room temperature, after which time they were fixed in 1% paraformaldehyde in phosphate‐buffered saline (PBS). All samples were run using an LSR II (BD Biosciences) and analysed using FlowJo software (Treestar, Ashland, OR, USA). The gate strategy can be seen in Supporting information, Fig. S1.

### Glucose and lipid uptake assays

Glucose uptake was assessed in CD27/CD45RA‐defined T cell subsets; PBMCs were incubated with anti‐CD3 (1 µg/ml) for 15 min at 37°C in complete media before being transferred into PBS for a further 15 min incubation prior to the addition of 100 µM 2‐NBDG. The PBMCs were then incubated with the 2‐(N‐(7‐nitrobenz‐2‐oxa‐1,3‐diazol‐4‐yl)amino)‐2‐deoxyglucose (2‐NBDG) together with anti‐CD3 for 30 min before being analysed by flow cytometry.

### Transwell migration assay

Human umbilical vein endothelial cell (HUVEC) monolayers were grown to confluence on Transwell membranes (Corning, New York, NY, USA) in the presence of 10 ng/ml IFN‐γ (R&D Systems, Abingdon, UK). PBMCs from healthy donors or T2Ds were placed in M199 medium (Sigma, Poole, UK) in the top well and the chemoattractant in the bottom well, which was 20% autologous donor sera. Cell migration was assessed after incubation at 37°C for 4 h; each condition was set up in duplicate Transwells. Migrated T cells were then collected from the top and bottom wells, respectively, and stained with phenotypical markers as described above.

### Statistical analysis

GraphPad Prism or ncss version 11 was used to perform statistical analysis. Statistical significance was evaluated using the Student’s *t*‐test or a repeated‐measures analysis of variance (anova) with the Tukey correction used for *post‐hoc* testing. Differences were considered significant when *P* < 0·05.

## Results

### The diabetic milieu alters the normal balance of T cell subsets

Chronic inflammation and immune dysfunction have been thought to play a role in the pathogenesis of diabetes. To investigate this relationship we examined differential leucocytes in individuals aged > 55 years with or without T2D, controlling for gender and age as a continuous covariate while excluding metastatic disease. We found a higher absolute white cell count, including neutrophils and eosinophils, but evidence of lymphopenia in the diabetic cohort of patients (Table 1). These data suggest that T2D patients have differential leucocyte counts that are characteristic of raised levels of systemic inflammation 20.

**Table 1:** Patient characteristics.

Characteristic	Non‐diabetic patients	Diabetic patients	*P*‐value
Number of individuals	1275	246	
Age (years)	65 (64–65)	65 (65–66)	
Body mass index (kg m^2^)	28·4 (28·2–28·7)	30·7 (30·1–31·2)	
Sex [female, *n* (%)]	677	86	
White cell count (cells 10^9 ^l^–1^)	7·3 (7·2–7·4)	8·0 (7·7–8·4)	<0·0005
Lymphocyte count (cells 10^9 ^l^–1^)	28·0 (27·4–28·5)	26·7 (25·5–27·9)	<0·05
Neutrophil count (cells 10^9 ^l^–1^)	61·1 (60·6–61·7)	63·1 (61·7–64·6)	<0·005
Eosinophils count (cells 10^9 ^l^–1^)	2·6 (2·5–2·8)	3·0 (2·7–3·3)	<0·05
Monocyte count (cells 10^9 ^l^–1^)	8·1 (7·9–8·3)	7·7 (7·5–8·0)	0·4
Platelets (cells 10^9 ^l^–1^)	263 (252–274)	258 (254–262)	0·16

Descriptive leucocyte data for healthy patients and patients with type 2 diabetes. Data were controlled for gender and age as a continuous covariate, patients with metastatic disease were excluded. All values mean proportion (95% confidence interval) or *n* (%).

**Figure 1:**
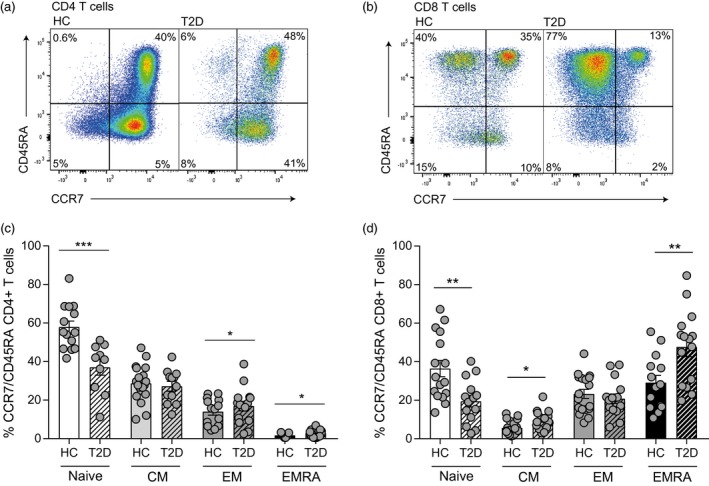
Elevated levels of T cell senescence in people living with type 2 (T2D) diabetes. The accumulation of senescent T cells in diabetes was defined using the markers C‐C chemokine receptor type 7 (CCR7) and CD45RA in T cells from patients with T2D and healthy controls aged > 55 years. Representative flow cytometry plots are shown for CD4^+^ (a) and CD8^+^ (b) T cells. Cumulative data of the phenotypical staining is shown for CD4^+^ (c) and CD8^+^ (d) CCR7/CD45RA‐defined T cell subsets. Data expressed as mean ± standard error of the mean (s.e.m.) of 16 healthy and 16 T2D donors. *P*‐values were calculated using a *t*‐test.

To determine how our observed changes in the white cell count related to the T cell population, we performed phenotypical analysis of CD4^+^ and CD8^+^ T cells in a subgroup of people living with T2D aged > 55 years recruited from the DARE database. We used flow cytometry to divide the T cell pool into four subpopulations based on their expression of surface receptors CCR7 and CD45RA. These subpopulations can be defined as naive (CCR7^+^CD45RA^+^), central memory (CM; CCR7^+^CD45RA^–^), effector memory (EM; CCR7^–^CD45RA^–^) and effector memory CD45RA re‐expressing cells (EMRA; CCR7^‐^CD45RA^+^) and represent successive stages of T cell differentiation 21, with the EMRA T cells being the senescent subset (Fig. 1a,b) 16. In people living with T2D we found a significant reduction in the naïve pool in both CD4^+^ and CD8^+^ populations; this was accompanied by a significant rise in the more differentiated EM and senescent EMRA populations of the CD4^+^ subset and CM and EMRA populations of the CD8^+^ T cells compartment compared to age‐matched controls (Fig. 1c,d). Overall, we show that T2D is a factor that drives the premature ageing of T cells, increasing T cell differentiation and senescence.

A wide variety of stressors including inflammation are known to cause premature senescence. As already stated, the T2D milieu, together with the presence of senescent cells, both contribute to the generation of an inflammatory environment. The presence of systemic inflammation is expressed here as a significantly raised neutrophil count (Table 1).

### Altered expression of surface chemokine receptors on T cells from people living with T2D

**Figure 2:**
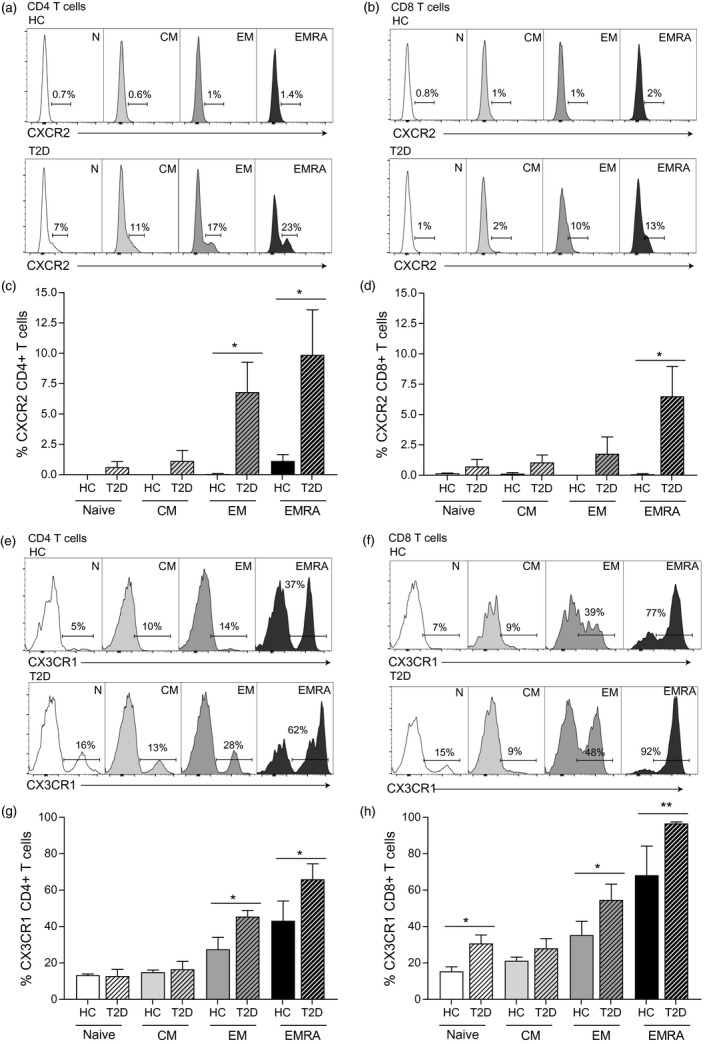
People living with type 2 diabetes (T2D) show increased proinflammatory chemokine receptor expression. Flow cytometry plots showing the expression of CXCR2 on both CD4^+^ (a) and CD8^+^ (b) CD45RA/CCR7 defined T cells from healthy controls and patients with T2D aged > 55 years. Together with graphs showing cumulative CXCR2 data from CD4^+^ (c) and CD8^+^ (d), T cells from nine healthy and nine T2D subjects. Representative CX3CR1 plots from both CD4^+^ (e) and CD8^+^ (f) T cell subsets and graphs for CD4^+^ (g) and CD8^+^ (h) T cells also from nine healthy and nine T2D individuals aged > 55 years. Graphs shows the mean ± standard error of the mean (s.e.m.) with *P*‐values calculated using a *t*‐test.

The vast effects of systemic inflammation on normal T cell physiology is largely uncharacterized, as a characteristic feature of chronic inflammation is the anomalistic infiltration of lymphocytes into non‐lymphoid tissues 22. We therefore examined the expression of migration‐associated chemokine receptors on T cells using flow cytometry from people living with T2D. We analysed the CXCR2 chemokine receptor, classically thought to be located on the neutrophil cell membrane and expressed scarcely in T cells 23. We found it to be significantly overly expressed in both the EM and EMRA populations of CD4^+^ (Fig. 2a,c) and the EMRA population (Fig. 2b,d) of CD8^+^ T cells of people living with T2D compared to healthy controls.

The CX3CR1 chemokine receptor, involved in the infiltration and proinflammatory polarisation of leucocytes, was increasingly expressed as T cells gained effector functionality (Fig. 2e–g). Furthermore, we observed a distinct increase in expression of CX3CR1 in CD4^+^ EM and EMRA populations (Fig. 2e,g) and the CD8^+^ naive, EM and EMRA T cell populations of people living with T2D (Fig. 2f,h). Taken collectively, these data suggest an abnormal promigratory phenotype of CD4^+^ and CD8^+^ T cells in T2D, although the extent of which this is attributed to chronic inflammation and or abnormalities in the serum glucose is unclear.

### T cells from people living with T2D accrue functionally impaired migration

**Figure 3:**
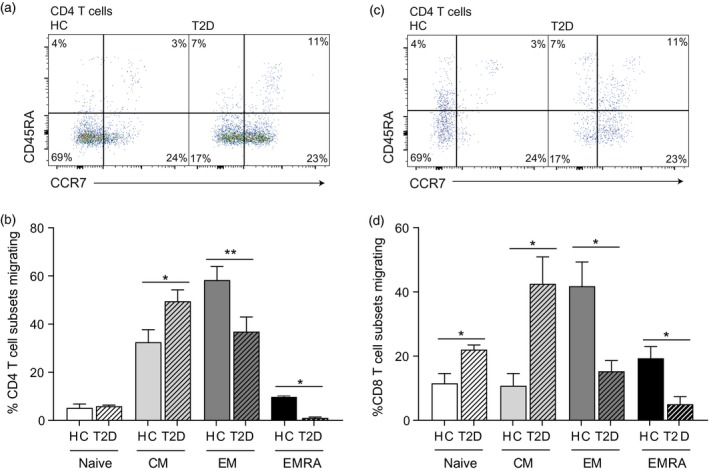
People living with type 2 diabetes (T2D) show impaired T cell migration. Flow cytometry plots showing the migration of CD4^+^ (a) and CD8^+^ (b) CD45RA/CCR7‐defined T cell subsets from healthy controls and patients with type 2 diabetes aged > 55 years through human umbilical vein endothelial cells (HUVECs) and their supporting Transwell filters. HUVECs were stimulated with 10 ng/ml interferon (IFN)‐γ for 24 h before peripheral blood mononuclear cells (PBMCs) were allowed to adhere and migrate for 4 h towards autologous serum. The percentage of each T cell subset migrating is represented in a graph for both CD4^+^ (c) and CD8^+^ (d) T cells. Graphs show the mean ± standard error of the mean (s.e.m.) of four healthy and four T2D individuals with *P*‐values calculated using a *t*‐test.

Migration plays an integral role in the normal immune function of lymphocytes. To functionally correlate our flow cytometry data, which suggest a promigratory phenotype in T cells isolated from people living with T2D, Transwell chemotactic assays were performed. HUVECs were activated using IFN‐γ and migration was assessed in response to 20% autologous serum (Fig. 3 a,b). We used autologous sera to replicate *in‐vivo* conditions more effectively rather than observing the migration in response to one chemokine. We found that the CD4^+^ CM subset and the N and CM CD8^+^ subsets from patients living with T2D showed increased migratory capacity (Fig. 3c,d). However, both the CD4^+^ and CD8^+^ EM and EMRA diabetic subsets showed significantly impaired migration (Fig. 3c,d). Given our chemokine phenotyping, together with diabetic sera containing a higher concentration of chemokines (data not shown), this was an unexpected result.

**Figure 4:**
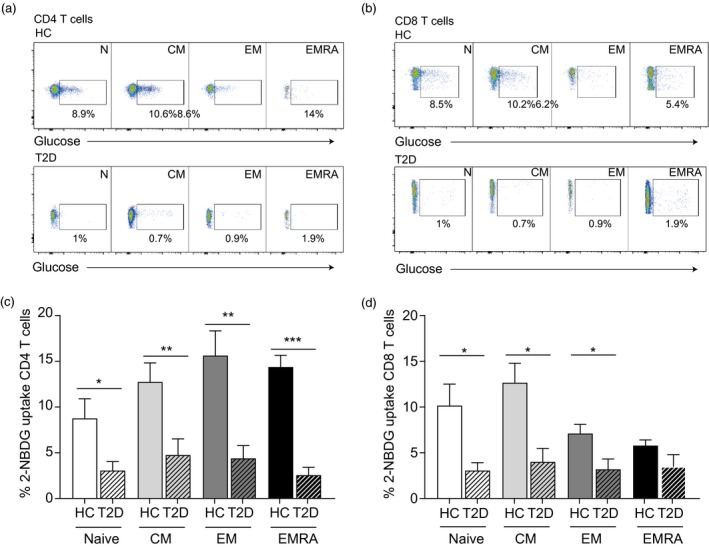
Impaired glucose uptake in T cells isolated from people living with type 2 (T2D) diabetes. Flow cytometry plots showing glucose uptake, assessed using the fluorescent glucose analogue 2‐NBDG in CD4^+^ (a) and CD8^+^ (b) CD45RA/CC27‐defined T cell subsets from healthy controls and patients with TD2 aged > 55 years. Graphs showing glucose uptake from CD4^+^ (c) and CD8^+^ (d) T cells from five healthy and five T2D subjects. Graphs shows the mean ± standard error of the mean (s.e.m.) with *P*‐values calculated using a *t*‐test.

In the context of a disease propagated by inefficient cellular glucose absorption, an abnormality in glucose uptake may limit T cell migration, despite a functional gain in promigratory receptors 24. We used the fluorescent glucose analogue 2‐NBDG in a glucose‐free buffer to test whether the impaired migration arose as a consequence of an energy deficit in the diabetic T cells (Fig. 4a,b). We found that all the CD4^+^ and CD8^+^ subsets displayed a lack of glucose uptake compared to the age‐matched T cells (Fig. 4c,d). This lack of glucose uptake probably generates an energy imbalance in the T cells from people living with T2D which gives rise to their decreased migratory ability, despite their up‐regulated chemokine receptors, and may be a cause of their functional impairment.

## Discussion

Since the seminal paper by Hayflick and Moorhead, evidencing the finite replicative ability of fibroblasts in culture 25, it is now known that cellular senescence can also be induced in response to different stressors such as oxidative stress or oncogenic activation 8. In addition to growth arrest, senescent cells adopt several unique identifying characteristics, including the accumulation of DNA damage foci, and an up‐regulation of cell cycle inhibitors and reactive oxygen species 26. Also prominent is the production of proinflammatory factors termed the senescence‐associated secretory phenotype (SASP), where senescent cells promote the development of chronic low‐grade sterile inflammation 27. It has been postulated that the presence of senescent cells may be the causative agent in the development and progression of T2D contributing to tissue dysfunction; however, the diabetic milieu could be permissive to the development of senescent cells 7. Whether or not senescence is the initial driver of T2D does not negate the existence of a detrimental feedback loop where metabolic dysfunction promotes cellular senescence that contributes to the worsening of disease 8.

Cellular senescence during diabetes has been well‐characterized in endothelial cells, renal mesangial cells, adipose‐derived stem cells and fibroblasts 28, 29, 30. However, very little work has been carried out in the immune compartment. Studies have eluded to an increase in senescent CD4^+^ T cells in young patients with T2D 31 and CD8^+^ senescent cells in older adults with T2D 32, although in both papers cells were defined as being senescent using only the loss of the co‐stimulatory marker CD28. While the loss of CD28 is often associated with senescence, CD28^‐^ T cells are a heterogeneous mix of two distinct T cell populations: EM and EMRA. Here we conclusively demonstrate that while people living with T2D exhibit lymphopaenia the remaining T cells present are the senescent EMRA subset of CD4^+^ and CD8^+^ T cells.

Senescent T cell have been well documented to be a proinflammatory subset 14 secreting their own unique array of proinflammatory products as part of their SASP 33, reinforcing both the senescent and inflammatory phenotype of these cells. The inflammatory environment of T2D, evidenced here as a measurable rise in serum markers of inflammation, further contributes to the generation of senescent T cells. Inflammation also changes the expression of chemokine receptors 34, and we found that senescent T cells from people living with T2D acquired migratory receptors not normally associated with T cells, such as CXCR2. The chemokine receptor CXCR2 is a typical feature of the neutrophil surface membrane, enabling their migration from the bone marrow to the periphery 35. The relationship between CXCR2 and senescence has previously been explored, CXCR2 knock‐out studies using fibroblasts have shown mitigation of both replicative and oncogene‐induced senescence while also diminishing the DNA damage response 36. Furthermore, ectopic expression of CXCR2 results in premature senescence via a p53‐dependent pathway 36. Therefore, CXCR2 appears to be vital in initiating and sustaining senescence; whether the increased expression of CXCR2 observed on T cells merely reinforces the senescent phenotype or plays a migratory role requires further investigation.

The ligand of the chemokine receptor CX3CR1, fractalkine, is a known mediator of chemoattraction, adhesion and activation of CX3CR1‐expressing cells 37. This migration axis has been considered to be the mechanism of lymphocyte migration through the endothelium of inflamed tissues, a prominent feature during chronic inflammation 38. Given the inflammatory nature of T2D we also monitored the expression of CX3CR1 on senescent T cells. We showed increased levels of chemokine receptor CXCR3 on late differentiated CD4^+^ and CD8^+^ T cells, as well as the CD8^+^ naive population of people living with T2D. Diet‐induced obesity, like T2D, is also associated with systemic inflammation 39. The preferential migration into inflamed non‐lymphoid sites associated with increases in CX3CR1 have also previously been demonstrated in CD4^+^ T cells of mice fed a high‐fat diet 40. We postulate that the combination of systemic inflammation and the abnormalities in the extracellular environment, including supraphysiological glucose levels, to be the cause of the increased chemokine receptor expression in T cells.

Despite the phenotypical changes in the late‐stage T2D T cell population, suggestive of an increased migratory potential, we paradoxically show an impaired functional migration in this subgroup of T cells. The inability of inflammatory terminal stage T cells to migrate suggests their sequestration in the peripheral circulation. T cell migration is centred around the production of adenosine triphosphate (ATP) during aerobic glycolysis to provide a rapid source of ATP for cytoskeletal rearrangement 41. Previous studies have shown that interference with the glycolytic pathway results in constrained migration of CD4^+^ and CD8^+^ T cells 42. We show a large reduction in the glucose uptake across CD4^+^ and CD8^+^ T cells in T2D, suggesting an insulin resistant phenotype in the circulating T cell pool. Various large cohort studies outline the increased morbidity associated with T2D compared to the non‐diabetic population 43, 44, 45. Despite our growing insight into the underlying pathogenesis of T2D complications, current therapies are aimed at optimizing glycaemic control. There is increasing evidence that direct T cell dysfunction during T2D is responsible, in part, for classical diabetic complications, including increased rates of infection and impaired wound healing 46, 47. As T cell migration is vital in both the control of infection and the wound‐healing process, we identify a functional impairment that may contribute to co‐morbid disease.

Overall, our results therefore demonstrate that an increased presence of senescent T cells has a detrimental impact on immune function during T2D. Given that many of the complications occurring during T2D have been difficult to treat through glucose control alone, targeting senescent cells may be a more effective treatment option.

## Disclosures

The authors have no conflicting financial interests.

## Author contributions

E. Y. M. L. and S. M. H. wrote the paper, designed and performed the experiments and analysed the data. E. C. C. and L. A. C. performed experiments and reviewed the manuscript. G. A. H., V. B., M. P., S. F. and G. A. H. provided the type 2 diabetic samples and reviewed the manuscript. G. L. A. analysed the data set as well as reviewing the manuscript.

## Supporting information


**Fig. S1.** Defined is the gating strategy used throughout the paper. Live cells were gated using forward and side scatter. The single cells were gated then the live cells, followed by CD4 and CD8 T cells. These were then subdivide into CCR7 and CD45RA.
